# Identification of Key Genes and Potential Therapeutic Targets in Sepsis-Associated Acute Kidney Injury Using Transformer and Machine Learning Approaches

**DOI:** 10.3390/bioengineering12050536

**Published:** 2025-05-16

**Authors:** Zhendong Zhai, JunZhe Peng, Wenjun Zhong, Jun Tao, Yaqi Ao, Bailin Niu, Li Zhu

**Affiliations:** 1 School of Information Engineering, Nanchang University, Nanchang 330031, China; 5701122158@email.ncu.edu.cn (Z.Z.); 5701123068@email.ncu.edu.cn (J.P.); 9109123044@email.ncu.edu.cn (W.Z.); 5714123051@email.ncu.edu.cn (J.T.); 5714123008@email.ncu.edu.cn (Y.A.); 2 School of Medicine, Chongqing University, Chongqing 400016, China; bniu@cqu.edu.cn

**Keywords:** sepsis, acute kidney injury, transformer, machine learning, diagnostic model

## Abstract

Sepsis-associated acute kidney injury (SA-AKI) is a life-threatening complication of sepsis, characterized by high mortality and prolonged hospitalization. Early diagnosis and effective therapy remain difficult despite extensive investigation. To address this, we developed an AI-driven integrative framework that combines a Transformer-based deep learning model with established machine learning techniques (LASSO, SVM-RFE, Random Forest and neural networks) to uncover complex, nonlinear interactions among gene-expression biomarkers. Analysis of normalized microarray data from GEO (GSE95233 and GSE69063) identified differentially expressed genes (DEGs), and KEGG/GO enrichment via clusterProfiler revealed key pathways in immune response, protein synthesis, and antigen presentation. By integrating multiple transcriptomic cohorts, we pinpointed 617 SA-AKI-associated DEGs—21 of which overlapped between sepsis and AKI datasets. Our Transformer-based classifier ranked five genes (*MYL12B, RPL10*, *PTBP1*, *PPIA*, and *TOMM7*) as top diagnostic markers, with AUC values ranging from 0.9395 to 0.9996 (MYL12B yielding 0.9996). Drug–gene interaction mining using DGIdb (FDR < 0.05) nominated 19 candidate therapeutics for SA-AKI. Together, these findings demonstrate that melding deep learning with classical machine learning not only sharpens early SA-AKI detection but also systematically uncovers actionable drug targets, laying groundwork for precision intervention in critical care settings.

## 1. Introduction

Sepsis-associated acute kidney injury (SA-AKI) is a critical complication of sepsis, carrying substantial mortality risk and frequently requiring prolonged intensive care [1,2]. Sepsis, defined as life-threatening organ dysfunction caused by a dysregulated host response to infection, triggers both inflammatory and immunosuppressive pathways that contribute to multi-organ failure [3]. Improving Global Outcomes (KDIGO) criteria define acute kidney injury (AKI) based on increases in serum creatinine or decreases in urine output [4]. Specifically, Stage 1 AKI is characterized by an increase in serum creatinine to 1.5–1.9 times baseline or an increase of ≥0.3 mg/dL (≥26.5 µmol/L), or a reduction in urine output to <0.5 mL/kg/h for 6–12 h [5]. Despite these criteria, SA-AKI diagnosis remains challenging due to heterogeneous pathophysiology and delayed biomarker specificity. Identifying novel biomarkers and integrating them with machine-learning-driven analytics (e.g., electronic health records or proteomic/transcriptomic data) could enable automated diagnosis, prognostication, and personalized therapeutic targeting.

Recent advances in biomarker discovery have led to the identification of novel candidates, including neutrophil gelatinase-associated lipocalin (NGAL), kidney injury molecule-1 (KIM-1), and the combination of tissue inhibitor of metalloproteinases-2 (TIMP-2) and insulin-like growth factor-binding protein 7 (IGFBP7). For example, NGAL and cystatin C demonstrate utility in early risk stratification, with cystatin C (cutoff ≥15.1 mg/L) showing exceptional accuracy in predicting persistent SA-AKI (AUROC 0.977) [6]. Importantly, emerging evidence has demonstrated that the inflammatory response induced by sepsis amplifies kidney damage via mechanisms like cytokine activation [7]. Pro-inflammatory cytokines such as IL-6 (>35 pg/mL) and TNF-α (>20 pg/mL) not only amplify systemic inflammation but directly compromise glomerular integrity through NF-κB-mediated endothelial activation, increasing renal vascular permeability. These mediators orchestrate immunometabolic crosstalk, with IL-6 shown to suppress renal tubular fatty acid oxidation while TNF-α upregulates mitochondrial ROS generation, establishing a self-perpetuating cycle of injury [8,9]. In recent years, the development of precision medicine has offered new perspectives for personalized treatment, where understanding the key genetic pathways involved in SA-AKI is essential to developing more effective therapeutic strategies with fewer side effects [10]. Precision medicine advances have revolutionized SA-AKI management by identifying critical pathogenic pathways and biomarkers, enabling targeted therapies.

Based on 403 screened studies focusing on AKI prediction models, machine learning algorithms have been increasingly applied to SA-AKI, with traditional logistic regression (LR) being the most commonly used (44% of 25 studies), followed by extreme gradient boosting (XGBoost, 20%), support vector machines (SVMs), light gradient boosting (LightGBM), recurrent neural network-long short-term memory (RNN-LSTM), and categorical boosting (CatBoost) [11,12,13,14,15,16]. These models primarily focus on the early identification (60%), prognostic prediction (32%), and subtype identification (8%) of SA-AKI. XGBoost demonstrated superior performance in multiple studies, achieving AUROCs of 0.821–0.954 for early AKI detection and mortality prediction, while RNN-LSTM showed exceptional accuracy (AUROC 1.000) in predicting acute kidney disease progression [17]. Key predictors included serum creatinine, lactate, blood urea nitrogen, and diabetes mellitus, with biomarker-enhanced models performing comparably to clinical variable-based models [18]. Despite these advancements, significant limitations persist. Over 80% of studies were retrospective and single-center, predominantly using databases like MIMIC-III/IV, limiting generalizability [13,16]. While biomarker integration (e.g., urinary peptides, gene signatures) could enhance interpretability and pathophysiological relevance, only 16% of studies incorporated such approaches [19]. Model interpretability remained challenging, with only 24% employing methods like SHAP or LIME to address the “black box” nature of machine learning [15]. Additionally, studies neglected long-term outcomes (e.g., CKD transition) and non-ICU populations, while overreliance on late biomarkers like serum creatinine hindered early detection [1,20]. Future research should prioritize multicenter prospective validation, biomarker-augmented interpretability frameworks, and clinical integration to improve model robustness and therapeutic targeting interventions for SA-AKI [19,20,21].

Sepsis-associated acute kidney injury (SA-AKI) remains a critical challenge in clinical settings, with limited early diagnostic tools and therapeutic options despite its significant impact on patient outcomes. In this study, we hypothesize that our machine learning models, particularly those leveraging a Transformer-based deep learning approach, can predict SA-AKI with greater accuracy by identifying key gene biomarkers from complex gene-expression profiles. The objectives of this research are threefold: (1) to develop an integrated AI-driven framework combining Transformer-based models with classical machine learning techniques to detect gene biomarkers associated with SA-AKI, (2) to assess the diagnostic potential of these biomarkers through rigorous feature selection and validation, and (3) to explore potential therapeutic targets by analyzing gene–drug interactions, thereby paving the way for precision interventions in SA-AKI management.

## 2. Materials and Methods

### 2.1. Diagram of Study Flow and Data Collection

**Figure 1:**
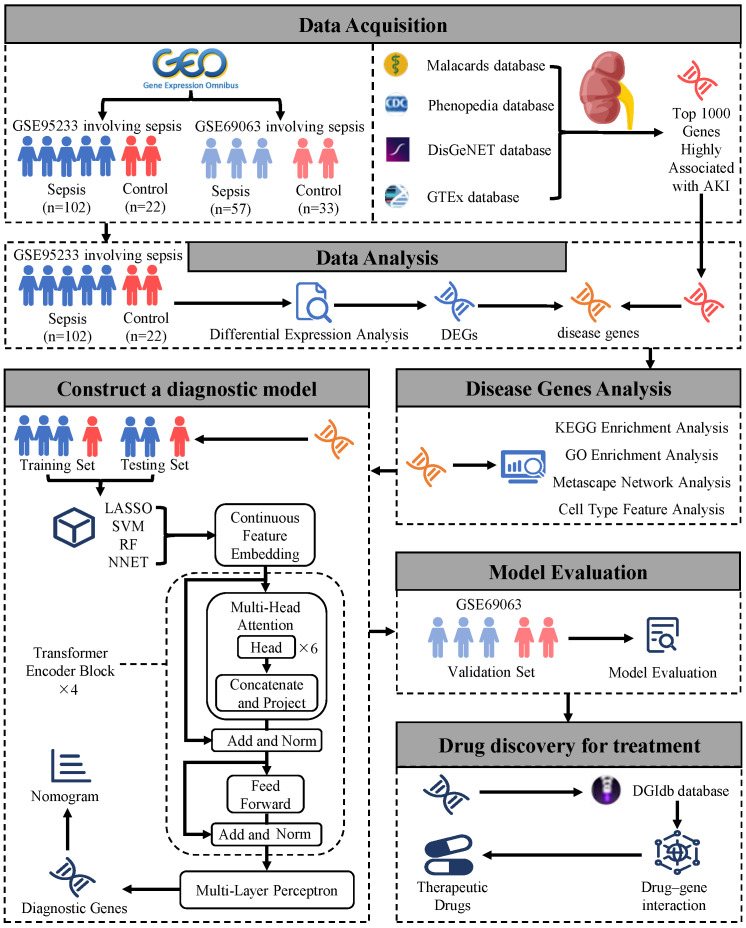
The whole workflow of this study.

Relevant datasets were selected from the NCBI Gene Expression Omnibus (GEO, https://www.ncbi.nlm.nih.gov/gds/) (accessed on 15 October 2024). Search keywords included “Sepsis” and “Homo sapiens”, and the data type was set to “Expression profiling by array” to obtain gene-expression data. After screening, the dataset GSE95233 was chosen as the primary dataset, and GSE69063 was selected as the validation dataset. The GSE95233 dataset is based on the GPL570 [HG-U133_Plus_2] Affymetrix Human Genome U133 Plus 2.0 Array and includes 124 samples, consisting of 102 sepsis samples and 22 healthy control samples. The GSE69063 dataset includes four disease phenotypes: allergic reaction, sepsis, trauma, and healthy controls. To meet the research requirements, 57 samples from septic patients and 33 samples from healthy controls were extracted, totaling 90 samples. The top 1000 genes highly related to acute kidney injury were selected from the GTEx (https://www.gtexportal.org/home/ (accessed on 15 October 2024)) [22], Malacards (https://www.malacards.org/ (accessed on 15 October 2024)) [23], Phenopedia (https://phgkb.cdc.gov/PHGKB/startPagePhenoPedia.action (accessed on 15 October 2024)) [24], and DisGeNET (https://disgenet.com/ (accessed on 15 October 2024)) [25] databases (accessed on 24 October 2024) and downloaded for further research analysis [26]. The whole workflow of this study in Figure 1.

### 2.2. Sepsis and AKI-Related Gene Analysis

Gene-expression data were normalized and annotated. Considering that multiple probes may map to the same gene symbol in the dataset, these probes were converted into gene symbols based on the annotation files of the platform. For probes mapping to the same gene symbol, the average expression value of these probes was selected as the gene’s expression level, thus obtaining the expression levels of each gene across different samples.

Differential expression analysis was performed using the limma package [27] in R (version 2024.12.1+563). This method employs a linear model to assess the expression differences of each gene between the sepsis group and healthy control group. During the analysis, a significance threshold was set as *p*-value < 0.05 and fold change (FC) greater than 1.2, i.e., FC > 1.2, as the criteria for selecting differentially expressed genes (DEGs). Using these criteria, genes with differential expression between the sepsis group and healthy control group were identified.

The DEGs were intersected with AKI-associated genes from public databases to identify shared sepsis-AKI disease genes. The overlapping genes between sepsis and AKI were identified and termed “disease genes”. The identification of these disease genes provides strong evidence for the relationship between sepsis and acute kidney injury, supporting the molecular correlation between them.

### 2.3. Gene Function Analysis

Gene Ontology (GO) functional analysis, which includes the terms Cellular Component (CC), Biological Process (BP), and Molecular Function (MF), is a powerful bioinformatics tool used to categorize gene expressions and their characteristics. KEGG pathway analysis is employed to identify the cellular pathways that may be involved in the changes in differentially expressed genes (DEGs). GO and KEGG pathway enrichment analyses of AKI and septic shock DEGs were conducted using the R package clusterProfiler [28]. A *p*-value of <0.05 was considered statistically significant.

### 2.4. Diagnostic Signature Machine Learning Analysis

To identify key diagnostic genes associated with SA-AKI, machine learning algorithms were employed to construct multiple classification models and perform feature selection using the DEGs identified in previous analyses. The disease-related genes identified in previous analyses were used as features, and corresponding gene-expression data were extracted for model construction. The dataset was randomly divided into a training set (70%) and a test set (30%) to ensure reliable model training and evaluation.

The glmnet R package [29] was used to implement the LASSO (Least Absolute Shrinkage and Selection Operator) algorithm for feature selection. LASSO applies L1 regularization to the model parameters, causing some coefficients to become zero, thus effectively selecting the most predictive gene features.

The SVM-RFE (Support Vector Machine Recursive Feature Elimination) algorithm was employed, using the SVM R package [30] for feature selection. This method recursively trains the SVM model, eliminating less important features and retaining those that have a greater impact on classification performance.

The randomForest R package [31] was used to implement the Random Forest (RF) algorithm. The RF algorithm constructs multiple decision trees and combines their results through voting, effectively identifying key gene features and providing an importance score for each gene.

The nnet R package [32] was applied to implement the artificial neural network (NNET) algorithm. The NNET model uses a multi-layer perceptron architecture to capture complex non-linear relationships between gene features and the target variable.

### 2.5. Diagnostic Signature Transformer Analysis

Building upon the machine learning approaches, the study also utilized the Transformer model to further enhance model performance and capture higher-order feature interactions. Unlike traditional machine learning algorithms, the Transformer model employs self-attention mechanisms to learn complex relationships between features, enabling it to capture dependencies that might be difficult to detect through conventional methods. The Transformer model, implemented using the transformer-pytorch library, was trained using the entire dataset, which included continuous features. This approach provided a deeper understanding of the genetic interactions involved in SA-AKI and facilitated more accurate identification of important diagnostic biomarkers.

For each of the four machine learning models (LASSO, SVM-RFE, RF, and NNET), the top 12 most important genes were selected. The intersection of these top genes from all four methods was then determined. These intersecting genes were subsequently input into the Transformer model, which prioritized the five most important genes for SA-AKI diagnosis. This comprehensive process led to the identification of a robust set of key biomarkers, enhancing the accuracy of SA-AKI diagnosis.

### 2.6. Model Construction and Evaluation

After selecting the key feature genes associated with SA-AKI, this study proceeded to construct diagnostic models based on the selected genes. These models aimed to distinguish between sepsis, acute kidney injury (AKI), and healthy control groups using the expression data of the selected genes.

To assess the diagnostic capability of the models, we calculated the AUC (Area Under the Curve) for the model and each feature gene. The AUC is a commonly used metric to evaluate the performance of binary classification models, with values closer to 1 indicating better diagnostic performance [33]. The AUC values for each gene were compared, and the accuracy and stability of the models were evaluated based on the combined results. To validate the clinical applicability of the constructed diagnostic models, we utilized the publicly available dataset GSE69063 as a validation set. This allowed us to assess the performance of the models on an independent sample set. By analyzing the prediction results from the validation set, we further confirmed the model’s ability to distinguish between sepsis and healthy controls, as well as between sepsis and AKI [34,35,36].

### 2.7. Drug Target Prediction

To identify potential drug targets, particularly for the treatment of SA-AKI, this study retrieved drug–gene interaction information related to the feature genes from the Drug Gene Interaction Database (DGIdb, https://www.dgidb.org/ (accessed on 15 October 2024)) [37].

We extracted the key feature genes and performed drug–gene interaction searches using the DGIdb database. DGIdb is a comprehensive resource for drug–gene information, offering detailed data on known and potential drugs, including drug–gene interactions, known targets, and mechanisms of drug action. These drugs include those already approved for other diseases as well as those showing potential efficacy in preclinical studies. Through drug–gene interaction analysis, we identified potential drugs targeting the key genes associated with sepsis and acute kidney injury, providing new directions for clinical treatment [38].

## 3. Results

### 3.1. Analysis of Genes Associated with Sepsis and AKI

**Figure 2:**
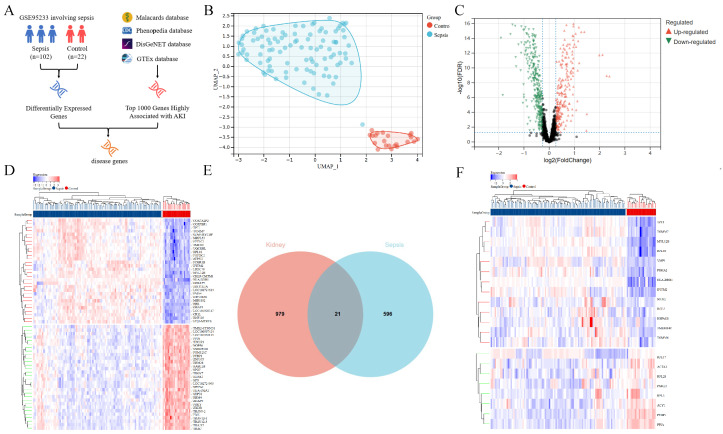
Analysis of genes associated with sepsis and AKI. (**A**) Identification process of key genes in sepsis and AKI. (**B**) UMAP plot of the GSE95233 Dataset. The UMAP analysis displays the distribution of sepsis (red circles) and control (blue circles) samples, with the x-axis representing UMAP1 and the y-axis representing UMAP2. The clear separation between the two groups indicates significant differences in gene-expression profiles. (**C**) Distribution of differentially expressed genes between the sepsis and control groups. The x-axis represents log2 fold change, and the y-axis represents −log10 FDR. Red dots indicate upregulated genes (248), and green dots indicate downregulated genes (369), with the threshold set at |log2FoldChange| > 1 and FDR < 0.05. (**D**) Heatmap of the top 30 DEGs between the sepsis and control groups. The x-axis represents samples (left: sepsis group; right: control group), and the y-axis lists the gene names of the top 30 DEGs. Red indicates upregulation, and blue indicates downregulation, with color intensity reflecting the magnitude of expression changes. (**E**) Overlapping genes between sepsis DEGs and key AKI genes, termed “disease genes”. The Venn diagram shows the overlap between sepsis DEGs (617 genes) and key AKI-related genes, identifying 21 shared genes defined as “disease genes”. (**F**) Heatmap of the 21 disease genes between the sepsis and control groups. The x-axis represents samples (left: sepsis group; right: control group), and the y-axis lists the 21 disease genes. Red indicates upregulation, and blue indicates downregulation, highlighting the expression differences between the two groups.

Through differentially expressed gene (DEG) analysis of the GSE95233 dataset, we first outlined the gene screening process associated with both sepsis and AKI (Figure 2A). UMAP (Uniform Manifold Approximation and Projection) analysis was then used to visualize the distribution of samples (Figure 2B), revealing a clear separation between sepsis and control groups without obvious outliers. A total of 617 DEGs were identified, including 248 upregulated and 369 downregulated genes (Figure 2C). The top 30 DEGs were further visualized using a heatmap to illustrate their expression patterns across samples (Figure 2D). Venn analysis showed that 21 genes were shared between sepsis DEGs and key AKI-related genes, defined as “disease genes” (Figure 2E). The expression profiles of these 21 disease genes between sepsis and control groups were subsequently displayed in a heatmap (Figure 2F).

### 3.2. Gene Function Analysis

**Figure 3:**
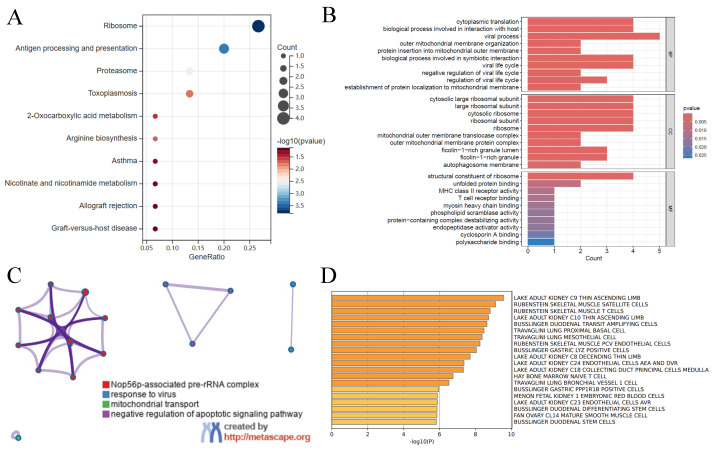
Enrichment analysis of disease genes. (**A**) KEGG pathway enrichment analysis of disease genes. (**B**) Top 10 GO terms, including Biological Process (BP), Cellular Component (CC), and Molecular Function (MF) of disease genes. (**C**) Enrichment network of disease genes obtained using the Metascape database, represented by cluster membership relationships. (**D**) summary of enrichment analysis in cell type signatures.

KEGG analysis showed that DEGs in SA-AKI were mainly enriched in pathways like “ribosome”, “antigen processing and presentation”, and “proteasome”. Other pathways such as “oxidative amino acid metabolism” and “Atoplasmosis” were also significantly enriched, suggesting involvement in immune response, protein degradation, and metabolic abnormalities (Figure 3A). GO enrichment analysis revealed that DEGs were enriched in “cytoplasmic translation”, “viral process”, and “protein insertion into mitochondrial outer membrane” in biological processes. In Molecular Function, genes were enriched in “structural constituent of ribosome” and “T cell receptor binding”. Cellular Component analysis showed enrichment in “outer mitochondrial membrane transport complex” and “cytoplasmic ribosomal subunit” (Figure 3B). Metascape network analysis classified 21 disease genes into functions related to “Nop56p-related prerRNA complex”, “response to virus”, “mitochondrial transport”, and “negative regulation of apoptotic signaling”, indicating their role in immune response, cellular stress, and cell death regulation (Figure 3C). Cell type signature analysis identified “adult kidney C9 thin ascending limb” and “adult kidney C10 thin ascending limb” cells as key in kidney repair and immune responses, with certain gut and lung cells also implicated (Figure 3D).

### 3.3. Diagnostic Signature Identification Analysis

**Figure 4:**
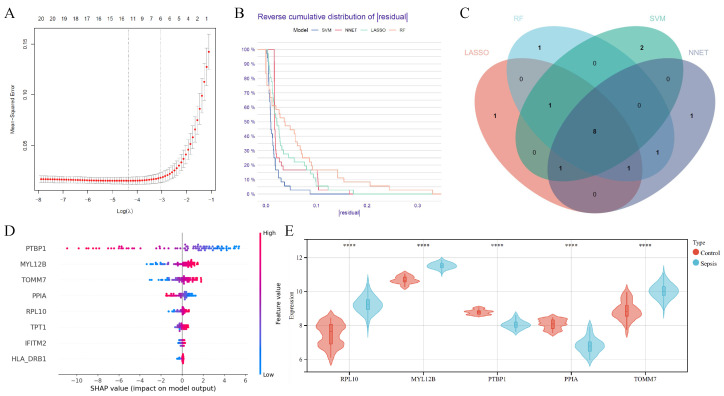
Analysis for identifying diagnostic signature. (**A**) Gene selection for diagnostic model construction via LASSO regression. The x-axis represents the λ value, and the y-axis represents cross-validation error. (**B**) Reverse cumulative distribution of residuals during the training process of the four machine learning models. (**C**) Overlapping genes selected by LASSO regression, SVM algorithm, random forest model and the artificial neural network. (**D**) SHAP values for feature importance in Transformer model for diagnosis. (**E**) Expression Levels of the 5 selected feature genes in the sepsis and control groups (**** indicates p<0.0001).

To identify diagnostic biomarkers for SA-AKI, four machine learning algorithms—LASSO, SVM-RFE, Random Forest (RF), and neural network (NNET)—were applied. The optimal λ parameter for LASSO regression was determined using cross-validation (Figure 4A). The performance of each algorithm during training was assessed through the reverse cumulative distribution of residuals (Figure 4B). Each algorithm selected the top 12 most informative genes based on their importance scores, as measured by dropout loss (where a smaller value indicates greater contribution to the model’s predictive power). This cutoff of 12 genes was chosen because an analysis of the dropout loss distribution across all models revealed a significant decline in predictive value beyond the 12th gene (Table S1). A sensitivity analysis, testing configurations of 10, 12, and 15 genes, further confirmed that 12 genes optimized model performance, as assessed by the Area Under the Curve (AUC) on the validation set GSE69063 (Figure S1). Each algorithm selected the top 12 most informative genes, and overlapping genes among the four methods were identified (Figure 4C). These candidate genes were subsequently used to construct a diagnostic model based on a Transformer architecture, and SHAP (Shapley Additive Explanations) analysis was performed to assess feature importance. Five genes with the highest SHAP values were identified as key diagnostic markers (Figure 4D). The expression levels of these five genes in sepsis and control groups are shown in Figure 4E, highlighting their differential expression and diagnostic relevance.

### 3.4. Diagnostic Model Validation

**Figure 5:**
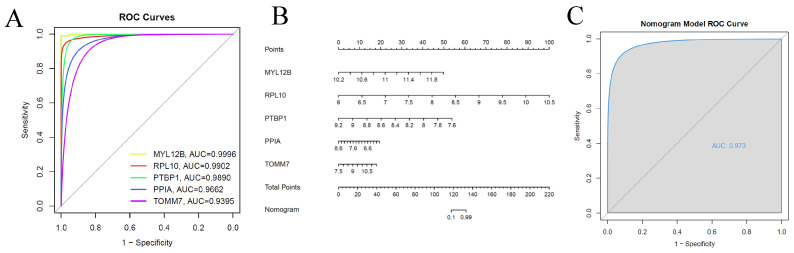
Validation of model for the diagnosis sepsis and AKI. (**A**) The ROC curve of the diagnostic signal showing each signature gene based on the datasets of GSE95233. (**B**) Norman plot constructed based on the 8 feature genes. (**C**) ROC curves of diagnostic signals showing the sensitivity and specificity of our models based on GSE69063.

The diagnostic capability of the five feature genes in distinguishing sepsis and healthy samples demonstrated good diagnostic value (Table S1). In our model, the AUC was 1.0, with MYL12B having an AUC of 0.9996, RPL10 an AUC of 0.9902, PTBP1 an AUC of 0.9890, PPIA an AUC of 0.9662, and TOMM7 an AUC of 0.9395 (Figure 5A). A nomogram was constructed based on the five feature genes, providing a visual representation of the diagnostic model. This nomogram facilitates the prediction of sepsis and AKI by summing the individual contributions of each gene (Figure 5B). A diagnostic model constructed based on these five genes was validated using the independent dataset GSE69063. This external validation yielded an AUC of 0.973 (Figure 5C). The corresponding ROC curve confirmed the model’s ability to accurately distinguish SA-AKI samples from healthy kidney samples, supporting the potential clinical utility of the identified biomarkers.

### 3.5. Drug–Gene Interaction Analysis

**Figure 6:**
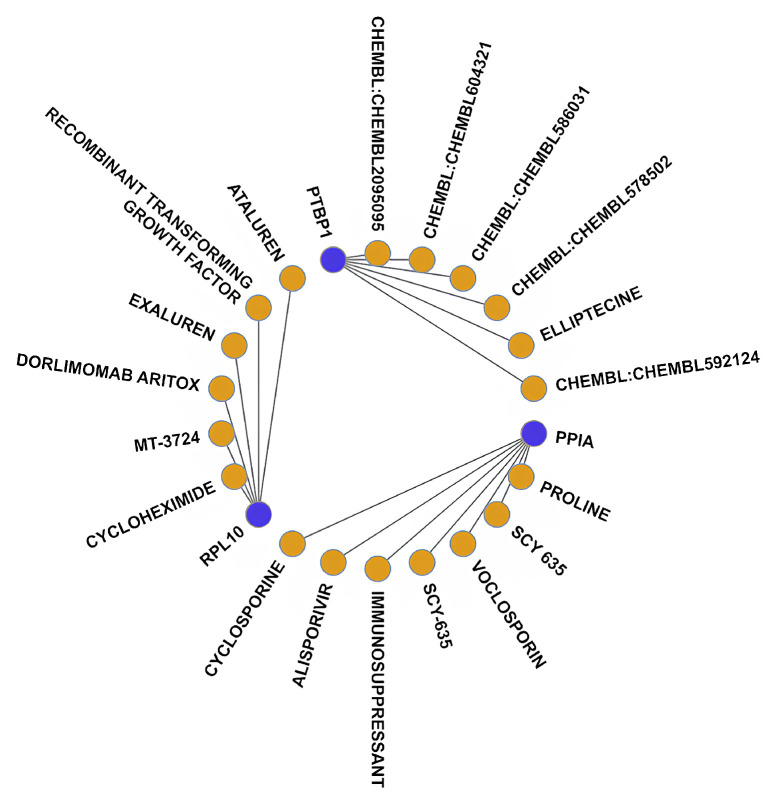
Drug–gene interaction diagram, blue circles represent signature genes and the yellow circles represent drugs.

To explore potential drug–gene interactions relevant to SA-AKI, we queried the DGIdb database using the 12 key genes identified by our machine learning models as critical to SA-AKI pathogenesis. This analysis identified 19 drugs with documented interactions with these genes, based on interaction confidence scores (>0.7) and established pharmacological data (Figure 6). These drugs include various immunosuppressants, antiviral agents, and some anticancer drugs, suggesting that they may play significant roles in regulating immune responses, inflammation, and apoptosis. After excluding the unapproved drugs from the initial list, 11 drugs remained, which may serve as potential treatments for sepsis and acute kidney injury (Table S3). Notably, some of these drugs, such as cyclosporine (associated with the PPIA gene), are well-documented nephrotoxins capable of causing AKI [39,40]. For example, cyclosporine interacts with PPIA (cyclophilin A), a protein involved in inflammatory pathways [40], yet its nephrotoxic profile, characterized by acute renal vasoconstriction and chronic structural damage [41], precludes its consideration as a therapeutic option for SA-AKI. Similarly, fluorouracil, linked to PTBP1 (an RNA-binding protein), emerged due to its regulatory effects on gene expression, though its clinical use is primarily in oncology and it has been associated with nephrotoxicity [42,43]. These associations, derived from computational analysis using machine learning models, highlight the complexity of drug–gene interactions, where such models can uncover connections reflecting both potential biological relevance and adverse effects, depending on the clinical context [44,45].

## 4. Discussion

This study introduces an innovative approach to identifying key diagnostic biomarkers for SA-AKI by integrating gene-expression analysis with advanced machine learning techniques, including the application of Transformer-based models in conjunction with traditional machine learning methods. Through a comprehensive multi-step process, we successfully identified pivotal feature genes that differentiate SA-AKI from healthy controls.

SA-AKI was interrogated via differential expression analysis, which uncovered 617 DEGs—including 248 upregulated and 369 downregulated genes. Subsequent Venn analysis identified 21 genes common to both sepsis and acute kidney injury, positioning them as potential biomarkers for SA-AKI. KEGG and GO pathway enrichment analyses further underscored the key biological processes involved: KEGG analysis demonstrated significant enrichment in immune response, protein synthesis, and antigen presentation pathways, while GO analysis revealed that these genes principally participate in immune response, cellular translation, and viral processes—pathways intimately linked to the pathogenesis of SA-AKI. To refine these biomarkers, an integrative feature selection strategy employing four machine learning algorithms (LASSO, SVM-RFE, RF, and NNET) was implemented, yielding eight consensus genes. Further enhancing model performance, a Transformer-based deep learning model was incorporated to capture complex nonlinear interactions, ultimately prioritizing five key genes as critical diagnostic biomarkers. The resultant diagnostic framework exhibited excellent performance in the validation set, affirming its reliability and predictive power. Moreover, drug–gene interaction mining via DGIdb prioritized 19 therapeutic candidates, offering novel avenues for intervention in SA-AKI.

The results of this study align with findings in the existing literature while also offering new perspectives. Key genes we identified, such as RPL10, MYL12B, and PPIA, have been shown to play significant roles in the mechanisms associated with sepsis and AKI. Specifically, RPL10, as part of the ribosome, plays a crucial role in translation elongation and the maturation of ribosomal subunits [46], a function that is supported by our findings linking immune response and protein synthesis pathways. Similarly, MYL12B is involved in regulating dynamic changes in the cytoskeleton and influences the cell division process by modulating the activity of myosin II [47], which correlates with the relationship between cell division and immune regulation observed in our study. PPIA promotes inflammation and cell signaling, playing a role in kidney injury [40], a finding supported by this study, suggesting its potential as a therapeutic target for sepsis and AKI.

In this study, we introduce a novel AI-driven framework that synergistically integrates Transformer models with machine learning algorithms (LASSO, SVM-RFE, Random Forest, and NNET) to identify diagnostic biomarkers and therapeutic targets for SA-AKI. This innovative methodology not only offers fresh insights into disease mechanisms but also combines differential gene-expression analysis, KEGG/GO pathway enrichment, machine-learning-based feature selection, and drug–gene interaction analysis to provide a comprehensive view of SA-AKI. The identification of five key genes and 19 potential therapeutic candidates furnishes valuable references for early diagnosis and targeted intervention.

Despite these advancements, our study has limitations. Primarily, it relies on public datasets (e.g., GSE95233) without the inclusion of clinical data, which constrains the clinical applicability and predictive power of our model. Future research should integrate clinical parameters within multicenter, large-sample studies to further assess the diagnostic and prognostic value of these biomarkers. Moreover, although KEGG and GO analyses have highlighted potential biological pathways, further in vitro and in vivo studies are required to elucidate the roles of these genes in immune responses and apoptosis. Overall, larger clinical investigations and experimental validations are essential to confirm these findings and explore their clinical applications.

## 5. Conclusions

The AI-driven integrative framework developed in this study identified five key diagnostic biomarkers (RPL10, MYL12B, TOMM7, PTBP1, and PPIA) for SA-AKI, achieving high diagnostic accuracy (AUC 0.9395–0.9996) across multicenter cohorts. Mechanistic investigation identified immune response and ribosomal pathways as core dysregulated modules, showing significant functional enrichment within SA-AKI hub genes. Systematic drug–gene interaction analysis revealed 19 mechanistically targeted compounds selectively engaging pathological networks for SA-AKI. This study provides a computational framework integrating biomarkers and therapeutic discovery, advancing the mechanistic understanding of sepsis-induced organ dysfunction. The conserved biomarkers uncovered by our approach offer new insights for developing precision diagnostics and tailored therapies in critical care settings.

## Data Availability

All data used in this study are publicly available from the GEO, GTEx, Malacards, Phenopedia, and DisGeNET databases.

## Supplementary Materials

The following supporting information can be downloaded at https://www.mdpi.com/article/10.3390/bioengineering12050536/s1, Figure S1. Validation of the nomogram model for the diagnosis of sepsis and AKI using different gene configurations on GSE69063. (A) 10 genes. (B) 12 genes. (C) 15 genes. Table S1. Dropout loss values of 21 genes in four machine learning models. Table S2. Descriptions and functions of the 5 feature genes used for diagnosis. Table S3. Target and indication of some drugs for the potential treatment of SA-AKI disease.

## Abbreviations

The following abbreviations are used in this manuscript:

SA-AKI	Sepsis-associated acute kidney injury
DEG	Differentially expressed gene
KEGG	Kyoto Encyclopedia of Genes and Genomes
GO	Gene Ontology
LASSO	Least Absolute Shrinkage and Selection Operator
SVM-RFE	Support Vector Machine Recursive Feature Elimination
RF	Random Forest
NNET	Artificial neural network
AUC	Area Under the Curve
DGIdb	Drug Gene Interaction Database
UMAP	Uniform Manifold Approximation and Projection
ROC	Receiver Operating Characteristic
TNF-α	Tumor Necrosis Factor-alpha
IL-6	Interleukin-6
BP	Biological process
CC	Cellular Component
MF	Molecular Function
SIRS	Systemic Inflammatory Response Syndrome
