# Supplementary material for: Identification of Key Genes and Potential Therapeutic Targets in Sepsis-Associated Acute Kidney Injury Using Transformer and Machine Learning Approaches
Source: Bioengineering (Basel). 2025 May 16;12(5):536. doi: 10.3390/bioengineering12050536 (PMC12108565; doi:10.3390/bioengineering12050536)
Supplement: Supplementary file 1 [file bioengineering-12-00536-s001.zip › bioengineering-3626799-supplementary.pdf]

Supplementary Information for

Identification of Key Genes and Potential Therapeutic Targets in Sepsis-Associated Acute Kidney Injury Using Transformer and Machine Learning Approaches

Table of Contents

Supplementary Figure S1. Validation of the Nomogram Model for the Diagnosis of Sepsis and AKI Using Different Gene Configurations on GSE69063. 1

Supplementary Table S1. Dropout loss values of 21 genes in four machine learning models ..2

Supplementary Table S2. Descriptions and Functions of the 5 Feature Genes Used for Diagnosis. 3

Supplementary Table S3. Target and Indication of some drugs for the potential treatment of SA-AKI disease. 4

Supplementary Figure S1. Validation of the Nomogram Model for the Diagnosis of Sepsis and AKI Using Different Gene Configurations on GSE69063. (A) 10 Genes. (B) 12 Genes. (C) 15 Genes.

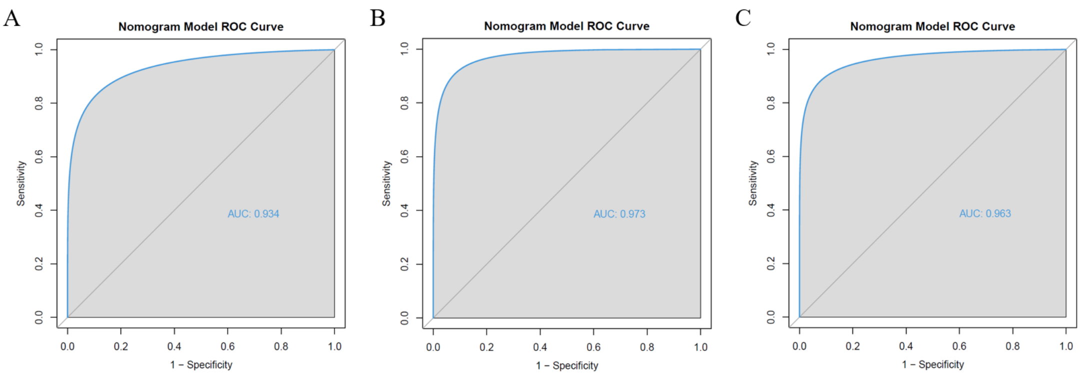

Supplementary Table S1. Dropout loss values of 21 genes in four machine learning models.

Gene	LASSO	NNET	RF	SVM
ACTA2	0.0581	0.0515	0.1022	0.0423
ACY1	0.0950	0.0505	0.1021	0.0425
HLA_DRB1	0.0901	0.1433	0.1258	0.0362
HSPA1B	0.1015	0.1554	0.0984	0.04506
IFITM2	0.0692	0.1536	0.1083	0.0312
MYL12B	0.0826	0.0518	0.1510	0.0266
NNMT	0.1020	0.0527	0.1625	0.0476
PPIA	0.0775	0.0786	0.1202	0.0305
PSMA2	0.0622	0.0513	0.1086	0.0234
PSME2	0.1108	0.1650*	0.1709	0.0551
PTBP1	0.0765	0.0601	0.1346	0.0283
RGL3	0.1128	0.1670*	0.1726	0.0573
RPL10	0.0842	0.1452	0.1341	0.0277
RPL17	0.0977	0.0543	0.1573	0.0238
RPL28	0.1145	0.1693	0.1741	0.0599
RPL3	0.1163	0.1712	0.1762	0.0617
TMEM14C	0.1049	0.1598	0.1648	0.0240
TOMM6	0.0588	0.1574	0.1627	0.0493
TOMM7	0.0712	0.0646	0.1065	0.0277
TPT1	0.0759	0.1520	0.1190	0.0266
VMP1	0.0599	0.0513	0.1557	0.0293

Supplementary Table S2. Descriptions and Functions of the 5 Feature Genes Used for Diagnosis.

Gene	Expression level	Description	Function
RPL10	High	Ribosomal protein L10, a component of the 60S ribosomal subunit.	Plays an important role in ribosome biogenesis and function, particularly in ribosomal subunit maturation, ribosomal subunit binding, and subunit rotation during translation elongation.
MYL12B	High	Encodes myosin light chain 12B, which forms an actin-myosin complex by binding with actin heavy chains.	MYL12B regulates the dynamic changes of the cytoskeleton, affecting cell morphology and movement. It plays a role in cytokinesis during cell division and may be involved in cell signaling processes.
PTBP1	Low	A gene encoding the polypyrimidine tract-binding protein.	1. Binds with lncRNA MIAT, induces the degradation of BECN1 mRNA, and inhibits autophagy activation. 2. Suppresses the protective effects of autophagy, exacerbating kidney damage. 3. Indirectly affects the intensity of the inflammatory response by regulating the stability of BECN1 mRNA and influencing the release of pro-inflammatory cytokines.
TOMM7	High	A component of the mitochondrial outer membrane translocase.	TOMM7 aids in the transport of precursor proteins and is essential for mitochondrial function, including respiration and energy metabolism. Mutations in TOMM7 disrupt mitochondrial protein expression and oxygen consumption.
PPIA	Low	Encodes peptidylprolyl isomerase A.	PPIA is involved in protein folding, translation regulation, and cell signaling. It helps maintain protein homeostasis and regulates protein translation efficiency. Secreted PPIA activates inflammation pathways (e.g., ERK1/2, p38 MAPK), promoting inflammation and tissue damage.

Supplementary Table S3. Target and Indication of some drugs for the potential treatment of SA-AKI disease.

Drug Name	Target	Indication
PROLINE	Proline dehydrogenase 1 mitochondrial	Hepatic encephalopathy
SCY-635	Cyclophilin A	Hepatitis C virus (HCV) infection
Voclasporan	Cyclophilin A	Hepatitis C virus (HCV) infection
Immunosuppressant	Multiple immune system targets	Organ transplantation
Alisporvir	HCV NS5A inhibitor	Hepatitis C
Cyclosporine	Calcineurin	Organ transplantation
Cycloheximide	Ribosome	Bacterial infection research
MT-3724	CD20	Leukemia and lymphoma
Dorlimomab Aritox	CD19	Leukemia and lymphoma
Exaluren	Collagen IV	Alport syndrome caused by nonsense mutations
Recombinant Transforming Growth Factor	TGF-β receptor	Diabetic foot ulcers, burns, pressure sores, and other tissue injuries
Ataluren	DAG1	Duchenne muscular dystrophy (DMD)
